# Pattern recognition receptor-mediated cytokine response in infants across 4 continents^⋆^^☆^

**DOI:** 10.1016/j.jaci.2013.09.038

**Published:** 2014-03

**Authors:** Kinga K. Smolen, Candice E. Ruck, Edgardo S. Fortuno, Kevin Ho, Pedro Dimitriu, William W. Mohn, David P. Speert, Philip J. Cooper, Monika Esser, Tessa Goetghebuer, Arnaud Marchant, Tobias R. Kollmann

**Affiliations:** a Department of Experimental Medicine, University of British Columbia, Vancouver, British Columbia, Canada; b Department of Pediatrics, University of British Columbia, Vancouver, British Columbia, Canada; c Department of Microbiology and Immunology, University of British Columbia, Vancouver, British Columbia, Canada; d Centro de Investigaciones FEPIS, Esmeraldas, Quininde, Ecuador; e Molecular and Biochemical Parasitology, Liverpool School of Tropical Medicine, Pembroke Place, Liverpool, United Kingdom; f Centro de Investgación en Enfermedades Infecciosas, Escuela de Biología, Pontificia Universidad Católica del Ecuador, Casilla, Quito, Ecuador; g Immunology Unit, Division of Medical Microbiology, Department of Pathology, NHLS and Stellenbosch University, Matieland, South Africa; h Department of Paediatrics, Centre Hospitalier Universitaire Saint-Pierre, Brussels, Belgium; i Institute for Medical Immunology, Université Libre de Bruxelles, Gosselies, Belgium

**Keywords:** Innate immunity, immune development, infectious disease, global, LPS, Lipopolysaccharide, MDP, Muramyl dipeptide, NOD, Nucleotide-binding oligomerization domain-containing protein, PCA, Principal-component analysis, PGN, Peptidoglycan, Poly I:C, Polyinosinic-polycytidylic acid, PRR, Pattern-recognition receptor, R848, Resiquimod, TLR, Toll-like receptor

## Abstract

**Background:**

Susceptibility to infection as well as response to vaccination varies among populations. To date, the underlying mechanisms responsible for these clinical observations have not been fully delineated. Because innate immunity instructs adaptive immunity, we hypothesized that differences between populations in innate immune responses may represent a mechanistic link to variation in susceptibility to infection or response to vaccination.

**Objective:**

Determine whether differences in innate immune responses exist among infants from different continents of the world.

**Methods:**

We determined the innate cytokine response following pattern recognition receptor (PRR) stimulation of whole blood from 2-year-old infants across 4 continents (Africa, North America, South America, and Europe).

**Results:**

We found that despite the many possible genetic and environmental exposure differences in infants across 4 continents, innate cytokine responses were similar for infants from North America, South America, and Europe. However, cells from South African infants secreted significantly lower levels of cytokines than did cells from infants from the 3 other sites, and did so following stimulation of extracellular and endosomal but not cytosolic PRRs.

**Conclusions:**

Substantial differences in innate cytokine responses to PRR stimulation exist among different populations of infants that could not have been predicted. Delineating the underlying mechanism(s) for these differences will not only aid in improving vaccine-mediated protection but possibly also provide clues for the susceptibility to infection in different regions of the world.

The first few years of life represent a period of marked susceptibility to infectious diseases.^1-3^ Such vulnerability reflects a state of age-dependent suboptimal immune-mediated protection in early life.^1,4,5^ Around the world, the Expanded Program on Immunization and similar regional or national programs direct the immunization of infants.^6,7^ These public health programs have greatly contributed to diminishing infectious mortality and morbidity in early life.^8^ Because the formulations and schedules of vaccination do not vary considerably among countries, these vaccination strategies rely on the notion that responses to vaccination would be similar among infants living in different regions of the world.^7,9,10^ However, it has become apparent that vaccine responses differ in infants from varying geographic regions.^11^ The underlying mechanisms leading to different vaccine responses in different populations remain largely unknown. This lack of understanding prevents optimization of infant vaccine responses. Because innate immunity directs adaptive immunity, we reasoned that the first step in identifying the mechanistic cause leading to variation in vaccine responses in infants from diverse regions of the world would be to determine whether differences in innate immunity exist among different populations from disparate regions. Several previous studies have described the ontogeny of the innate pattern recognition receptor (PRR) response in infants from different geographical regions.^5^ We set out to contrast the PRR response to stimulation of infants across 4 continents (Africa, North America, South America, and Europe) by using a highly standardized, stringently controlled innate immune phenotyping platform, ensuring the same experimental setup for all sites. We found significant differences in innate immune responses to PRR stimulation among infants from different populations.

## Methods

### Ethics statement

This study was conducted according to the principles expressed in the Good Clinical Practice Guidelines, and the Declaration of Helsinki. This study was approved by the University of British Columbia Ethics Board (protocol: H11-01423). In addition, each site involved had obtained ethics approval in its respective research center. Informed written consent from the next of kin, caregivers, or guardians on the behalf of the minors involved in this study was obtained for all study participants.

### Participant recruitment and enrollment

This study compared infants aged approximately 2 years from 4 different sites: Vancouver, Canada; Brussels, Belgium; Quininde, Ecuador; and Cape Town, South Africa. Canadian subjects were recruited from a pool of healthy infants participating in other ongoing research studies at the University of British Columbia.^12^ Subjects in Belgium were part of a pilot study for a larger urban-based birth cohort study established at St Pierre Hospital (Brussels) in collaboration with the Institute for Medical Immunology. Infants from Ecuador were recruited within a rural-based population cohort study.^13^ South African infants had been enrolled in an urban-based birth cohort established at Stellenbosch University.^14^ A subject was included in the study if the infant was considered healthy on the basis of a history-driven health assessment. Subjects were excluded from the study if they had met 1 or more of the following criteria: significant chronic medical condition, immune deficiency, immunosuppression by disease or medication, cancer, bone marrow or organ transplantation, receipt of blood products within 3 months, bleeding disorder or major congenital malformation, or genetic disorder. Infants born to HIV-positive mothers were also excluded.

### Blood collection

Given that one of the major roles of the innate immune system is sensing environmental changes,^15,16^ technical artifacts can easily plague innate immune assessment.^17^ We thus implemented an experimental approach with stringent focus on quality control and assurance. Every step of the experiments was standardized and controlled across all sites. All materials and reagents from blood draw to final analysis were tested to ensure absence of innate immune activation substances as previous work had shown lot-dependent variation.^17,18^ All blood draws were performed in a hospital by a trained phlebotomist; the majority of the samples were collected from the arm, with some from the neck. Peripheral blood (3-5 mL) was drawn via sterile venipuncture into vacutainers containing 143 units of sodium-heparin (Becton Dickinson [BD] Biosciences, San Jose, Calif, catalog no. 8019839). Blood samples were kept at room temperature and processed within hours of blood draw as described previously.^17,18^

### Toll-like receptor stimulation and blood culture

Master mixes of all reagents were made in quantities adequate for the entire study, frozen, and shipped under monitored conditions to all the 4 sites. The same person (K.S.) performed all aspects of the experiments at all sites by using our well-established robust, validated, and quality-controlled innate immune phenotyping protocol.^12,17-20^ In brief, deep 96-well (VWR, Mississauga, Ontario, Canada) source plates with each well containing 1.3 mL of a specific Toll-like receptor (TLR) ligand, were prepared by using sterile procedures under a laminar airflow hood. A total of 22 μL from each well of the source plate was dispensed into each well of recipient 96-well round-bottom polystyrene plates (Corning, Corning, NY) by using the Evolution P3 Precision Pipetting Platform (Perkin Elmer, Waltham, Mass). Recipient plates were sealed with sterile aluminum plate sealers and frozen at −80°C until use.

The 96-well plates contained the following TLR ligands with specified concentrations and specifically targeted PRR: PAM3CSK4 (PAM; TLR2/1; InvivoGen, San Diego, Calif) at 1 μg/mL; polyinosinic-polycytidylic acid (Poly I:C; TLR3; GE Healthcare, Fairfield, Conn) at 100 μg/mL; lipopolysaccharide (LPS; TLR4, InvivoGen) at 10 ng/mL; resiquimod (R848; TLR7/8, InvivoGen) at 10 μM; peptidoglycan (PGN; nucleotide-binding oligomerization domain-containing protein 1/2 [NOD1/2], InvivoGen) at 10 μg/mL; muramyl dipeptide (MDP; NOD 2, InvivoGen) at 0.1 μg/mL; and media alone. All TLR ligands were diluted in RPMI medium to obtain the desired concentration.

Whole blood was diluted 1:1 with sterile prewarmed RPMI 1640, and 200 μL of the diluted blood was added to each well of the premade plates containing the specific TLR ligands. Blood was incubated for 24 hours, after which plates were centrifuged at 600*g* and subsequently 100 μL of the supernatant was removed and frozen at −80°C for multiplex assay analysis later. Samples were shipped on dry ice via World Courier, Inc, with a temperature monitor in each shipment ensuring maintenance of the desired temperature (−80°C). Samples were stored at −80°C in the central analysis site (Vancouver, Canada), and were all run within 12 months of collection.

### Cytokine measurement

Supernatants were thawed at room temperature and assayed by multiplex assay technique (Luminex: Upstate/Millipore “Flex Kit” system) by using the high-biotin protocol with overnight incubation at 4°C. The levels of the following cytokines were measured: IFN-α2, IFN-γ, CXCL10, IL-12p70, IL-12p40, IL-6, TNF-α, IL-1β, CXCL8, CCL3, CCL4, and IL-10. Samples were diluted 1-to-1 (or 20-, 80-, or 150-fold) with RPMI 1640 as needed to fall within the standard curve. Beadlytes, biotin, and streptavidin-phycoerythrin were used at half the manufacturer’s recommended concentrations. Assays were read by using Luminex 200 Total System (Luminex, Austin, Tex) running either the Bio-plex (Bio-Rad, Hercules, Calif) or the MasterPlex (MiraiBio, San Francisco, Calif) software, and the downstream analysis was performed by using Excel (Microsoft) and an in-house database.

### Human IL-23 ELISA

To determine the IL-23 concentration, filtered supernatants were diluted 1:4 in diluent contained in the human IL-23 (p19/p40) ELISA kit (eBioscience, San Diego, Calif), and assays were performed according to the manufacturer’s specifications. Plates were read at 450 nm with 570 nm subtraction on a SPECTRAmax Plus. A 4-parameter sigmoid logistic curve was used to generate the standard curve.

### Statistical analysis

Kruskal-Wallis analysis was performed to compare the 4 sites for significant variance among the median cytokine concentrations. Bonferroni test was applied to correct for multiple comparisons. Dunn’s posttest was used to determine which of the sites contributed to the significant differences. Statistical analysis was conducted in Prism Version 6 (GraphPad Software).

### Principal-component analysis

To visualize the data in an intuitive fashion, we plotted the data by using principal-component analysis (PCA). The cytokine data were log-transformed and then subjected to PCA by using GINKGO: Multivariate Analysis System.^21,22^ The data were plotted by using Tableau visualization software (Tableau Software, Inc, Seattle, Wash). Because of low sample volume, IL-23 could not be assessed for each of the enrolled subjects; the IL-23 data were thus not included in the principal-component cluster analysis but were included in the box-plots and statistics.

### *Z*-score analysis

The World Health Organization anthropometric calculator was used to determine each participant’s individual *z* score (WHO Anthro version 3.2.2).^23^

## Results

### Cohort characteristics

We selected 4 populations that differ in many of the elements presumed to be relevant for variation in risk for infection or vaccine responses. Most importantly genetic variation among the hosts and differences in environmental exposure such as residence in resource-poor versus resource-rich settings. We chose to study innate immunity in infants aged 2 years to ensure that all had completed locally recommended infant vaccinations (see Fig E3 in the Online Repository at www.jacionline.org). The characteristics of the study population at the time of sample collection are described in Table I. Based on the WHO Child Growth Standards, the mean weight-for-age *Z* score, length-for-age *Z* score, and weight-for-length *Z* score of each subject in all cohorts were within less than 2 SDs of the mean (Table I). This indicated that the infants in our cohorts were within the average range for normal child growth standards.^24,25^ Furthermore, all infants were healthy based on clinical history taken at the time of sample collection.

### Innate cytokine responses

We chose 13 cytokine target read-outs to broadly cover the most important functional categories: innate cytokines supporting T_H_1-type adaptive immunity (IFN-α, IFN-γ, CXCL10, and IL-12p70), innate cytokines supporting T_H_17-type adaptive immunity (IL-12p40, IL-6, and IL-23), proinflammatory cytokines (TNF-α and IL-1β), chemokines (CXCL8, CCL3, and CCL4), and the regulatory cytokine IL-10. There were no significant differences in response between males and females (data not shown); thus the sexes were pooled for analysis for each site.

**Fig 1:**
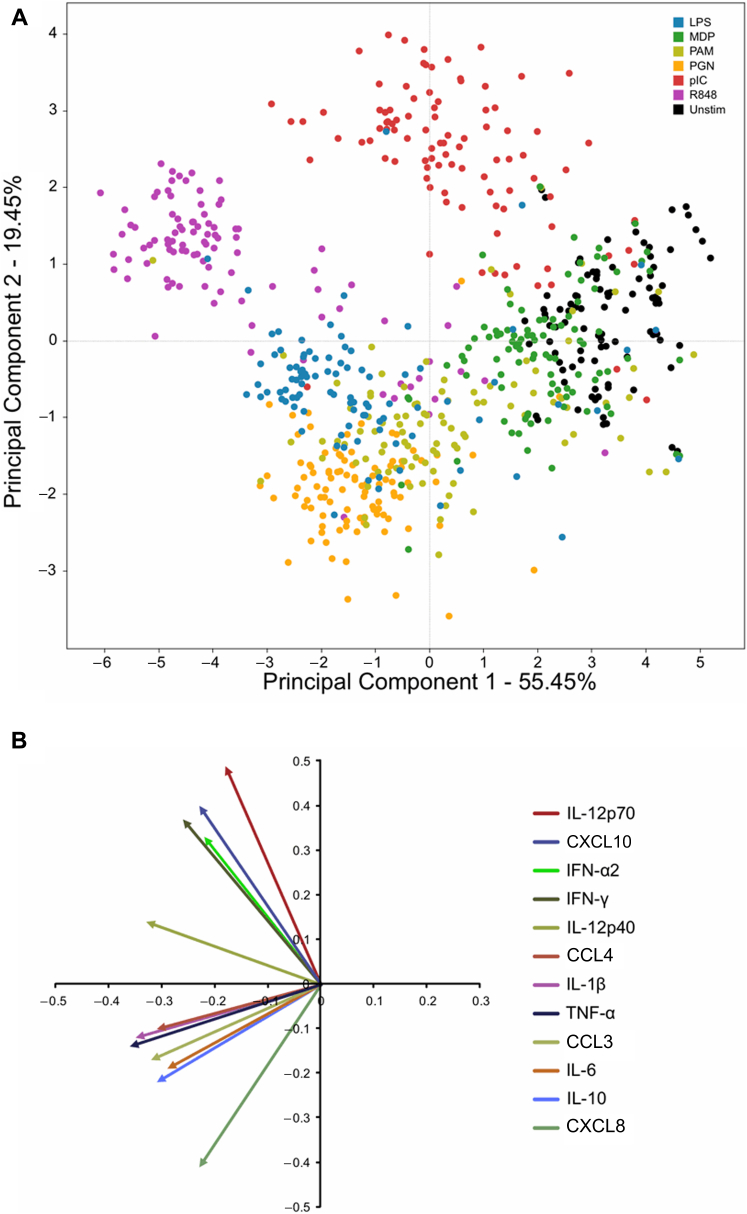
PCA ordination of the innate immune response for all subjects measured by cytokine secretion in response to PRR agonists. **A,** The variance in cytokine response (12 dimensions) to all ligands. Each *color* represents a ligand, while each *dot* represents 1 subject. **B,** Eigenvectors show the particular correlations of individual cytokines to the ordination of the PCA in *A*.

PCA (Fig 1) allowed us to compress the many dimensions (12 eigenvectors, each representing 1 cytokine) following response to all 7 PRR agonists for visual analysis.^22,26^ In Fig 1, each color represents a ligand and each dot represents 1 infant for a particular stimulatory condition. The percentage of PCA1 (55.45%) and PCA2 (19.45%) contributing to overall variance between subjects and stimuli is depicted on the x-axis and the y-axis, respectively. The primary component separating data points in Fig 1 (ie, principle component 1 [PC1]) appears to be the overall strength of the stimulation, as the weakest stimulant (unstimulated samples) clusters furthest to the right while the overall strongest stimulant (R848) clusters furthest to the left. PC2, however, separates the clusters on the basis of the PRR location; that is, endosomal PRR (TLR3 and TLR7/8) responses cluster higher on the axis, while cell surface and cytoplasmic PRR (TLR2/1, TLR4, and NOD) responses cluster lower down. The eigenvectors of the PCA are shown in Fig 1, *B*. This allows further delineation of contributors for the clustering along PC2, in that the endosomal TLR-driven clusters (ie, those located higher up in the plot) are largely composed of T_H_-1 supporting innate cytokines (IFN-α, IL-12p70, and IFN-γ) while the cell-surface TLR- and NOD-driven clusters (ie, those clustering in the lower left quadrant) are composed of the proinflammatory cytokines (TNF-α, IL-1β, etc). This pattern is also consistent with the known function of PRRs, in that endosomal PRRs mainly recognize intracellular pathogens and their activation leads to the production of innate cytokines supporting cell-mediated T_H_1-type immunity (ie, IFN-α and IL-12p70).^5,27^ Visual analysis indicated that the largest variance between samples was determined by the type of PRR stimulation (Fig 1). Furthermore, the fact that the PRR-induced responses led to similar clustering for most infants from all 4 sites suggests that basic biological mechanisms functioning in all populations represented the strongest component contributing to clustering.

### Endosomal TLR responses: South African infants underresponded

#### TLR7/8 (R848)

**Fig 2:**
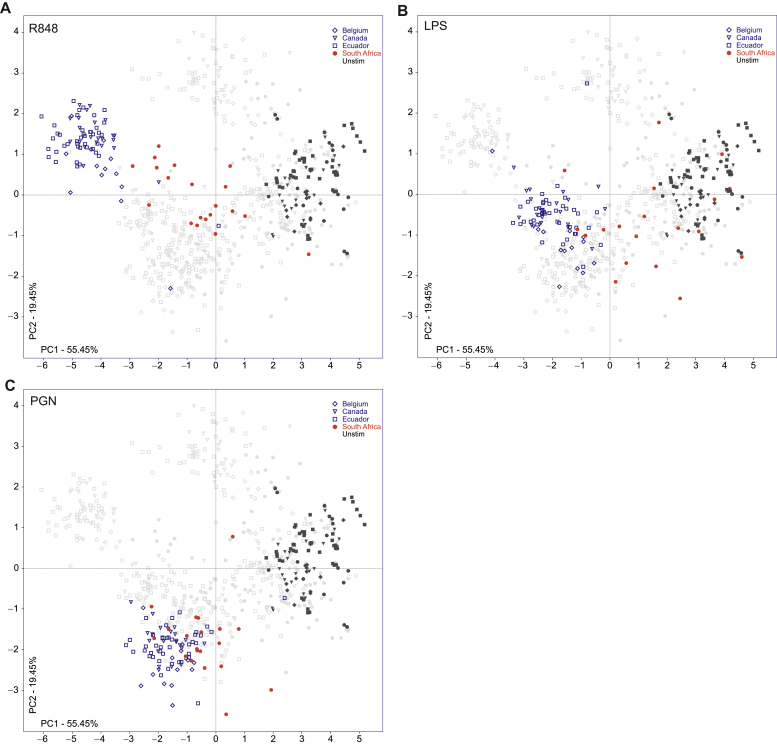
Innate immune response to PRR stimulation. PCA ordination of the R848 response is depicted in panel **A,** LPS in panel **B,** and PGN in panel **C**. Each *dot* represents 1 subject, *symbol* represents a site, and *color* represents the stimulation (*open* [Belgium, Canada, and Ecuador], *red* [South Africa] for given stimulation).

In Fig 2, *A*, we highlighted responses of all 4 cohorts to R848 (a TLR7/8 ligand) stimulation. The Belgian, Canadian, and Ecuadorian responses tightly clustered together at the upper left-hand quadrant of the ordination, clearly apart from the unstimulated samples. The response of infants in the South African cohort localized as a distinct and separate cluster between the Belgian-Canadian-Ecuadorian stimulated cluster and the cluster of all the unstimulated samples. Compared with the other geographic cohorts, this pattern suggests that South African infants respond differently to stimulation with R848.

**Fig E1:**
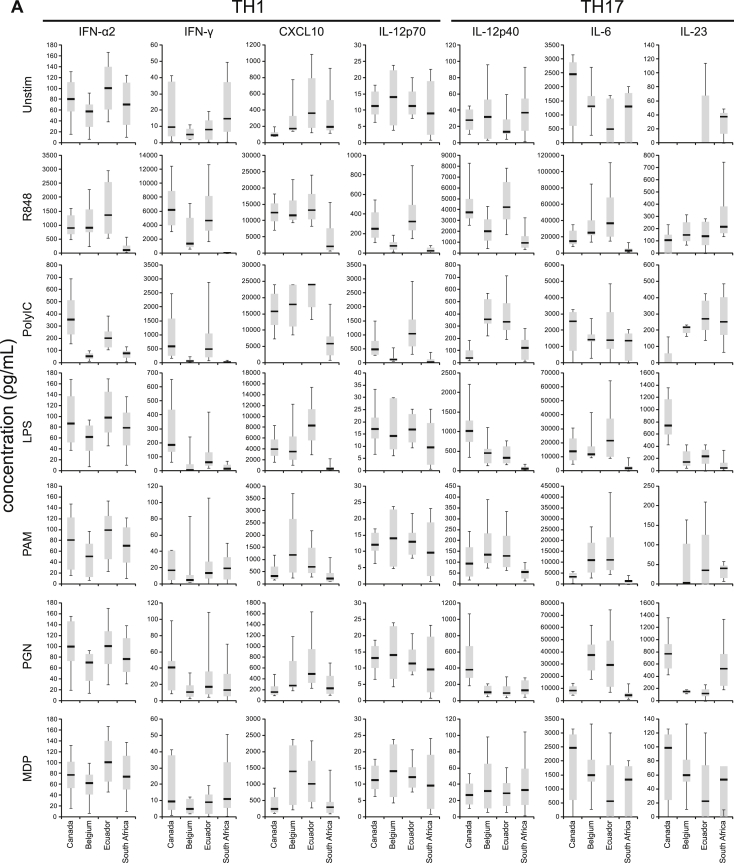

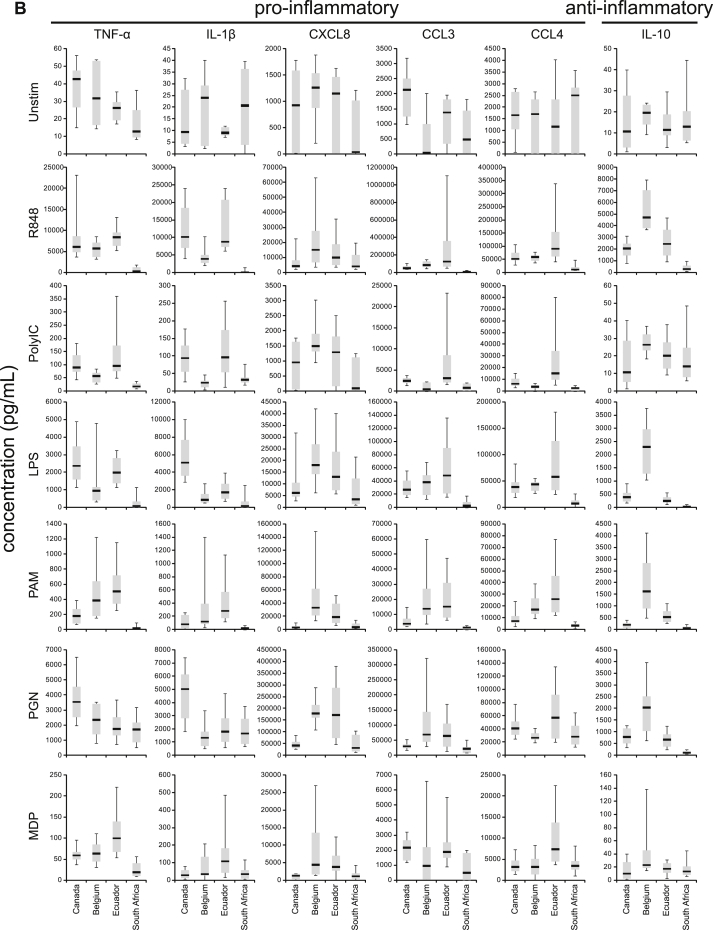
Cytokine production comparison for all 4 sites per cytokine and stimulation. Box-whisker plots, with the median highlighted for each site (the error bars = 90% CI). **A,** The production of T_H_1- and T_H_17-supporting innate cytokines. **B,** The production of the proinflammatory cytokines and chemokines, as well as the regulatory cytokine IL-10.

We also contrasted the production of individual cytokines between populations (see Fig E1 in the Online Repository at www.jacionline.org). Kruskal-Wallis analysis (Table II) revealed that with the exception of CXCL8, all cytokines produced in response to TLR7/8 stimulation were detected at significantly different levels among sites. Dunn’s posttest (Table II) further revealed that South African infants’ responses were solely responsible for the significant variation among sites, with South African infants responding consistently lower than infants from the other sites. For T_H_1-supporting innate cytokines, production in South African versus Ecuadorian and Canadian infants differed for all, and South Africa versus Belgium for most cytokines. For T_H_17-supporting innate cytokines, differences in production between South African versus Ecuadorian infants were consistently present, while IL-12p40 production displayed a significant difference between South African and Canadian infants’ responses, and IL-6 between South African and Belgian subjects. For the proinflammatory cytokines, significant difference between sites originated between South Africa and all other sites. Production of the proinflammatory chemokines was also significantly different between all sites, with South African infants producing less than Belgian, Canadian, or Ecuadorian infants. Furthermore, the antiinflammatory cytokine IL-10’s median concentration detected following TLR7/8 stimulation was lowest in South African infants, with Belgian infants producing the most.

#### TLR3 (Poly I:C)

**Fig E2:**
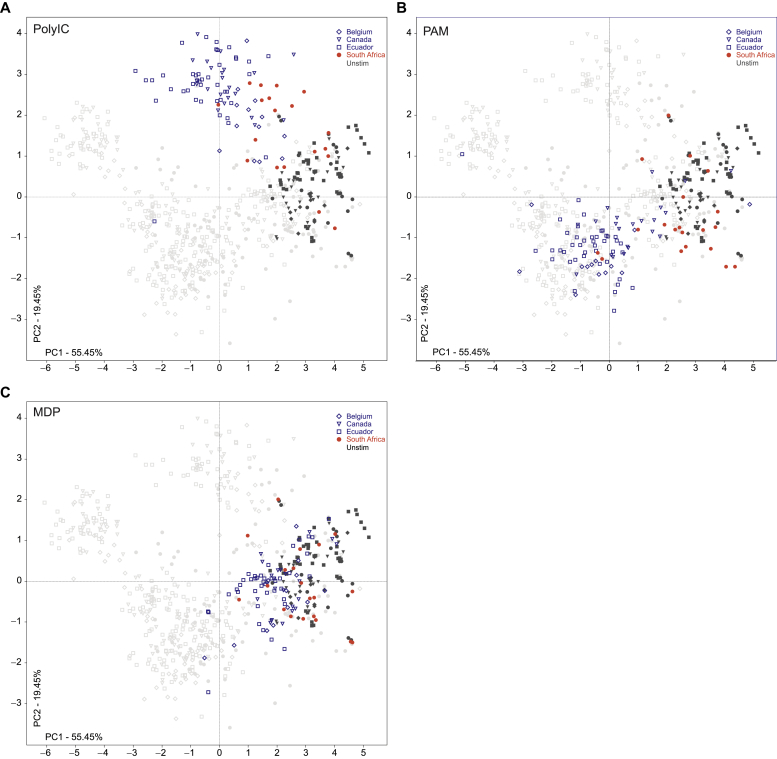
Innate immune response to PRR stimulation. PCA ordination of the Poly I:C response is depicted in panel **A,** PAM in panel **B,** and MDP in panel **C**. Each *dot* represents 1 subject, *symbol* represents a site, and *color* represents the stimulation (*open* [Belgium, Canada, and Ecuador], *red* [South Africa] for given stimulation).

**Fig E3:**
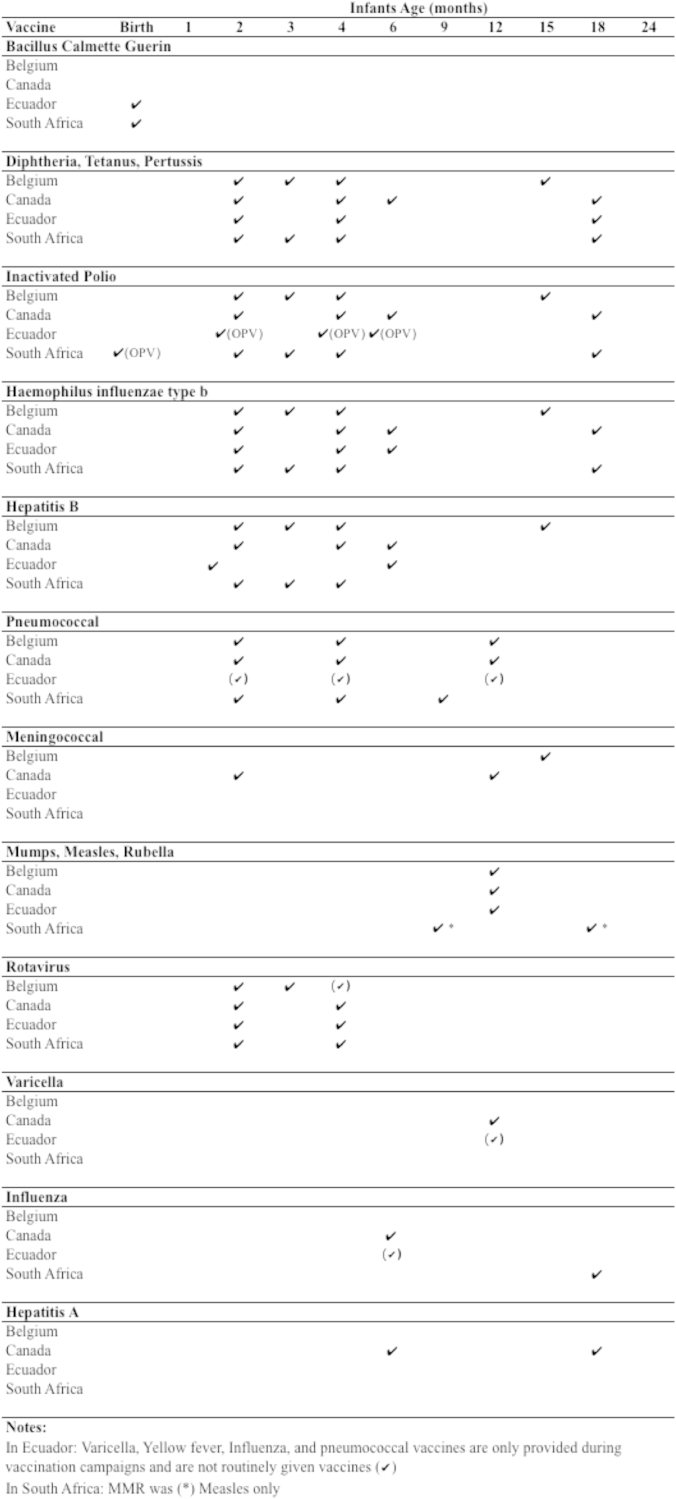
A simplified vaccine schedule for infants at each site.

PCA analysis of Poly I:C (a TLR 3 ligand) stimulation also led to similar responses at all sites except South Africa (see Fig E2, *A*, in the Online Repository at www.jacionline.org). South African infants produced low T_H_1-supporting and proinflammatory cytokines. However, the magnitude of the difference in cytokine production between the response of South African subjects and those from the other sites was not as large following TLR3 stimulation as it was following TLR7/8 stimulation. For example, the median response of the Belgian cohort located marginally above the South African cohort. Furthermore, Ecuadorian subjects stimulated with Poly I:C appeared to form 2 separate clusters, one grouping above and another below the main cluster composed of Belgian and Canadian subjects. The separation of the Ecuadorian subjects into 2 clusters is also reflected in the larger CI of median cytokine concentrations depicted in Fig E1.

Statistical analysis (Table II) revealed that cytokines were produced at significantly different levels among the 4 sites following TLR3 stimulation, with the exception of IL-6, CXCL8, and IL-10. Dunn’s posttest of T_H_1-supporting and proinflammatory cytokine production revealed that the variation mainly originated from South African infants. Production of IL-10 following Poly I:C stimulation was not above background for subjects from any of the 4 sites.

### Responses to cell surface TLRs: South African infants underresponded

#### TLR4 (LPS)

LPS stimulation of TLR4 resulted in clustering of response for infants from all sites except from South Africa (Fig 2, *B*). This illustrates that compared with subjects from other sites, whole blood from South African infants also underresponded to TLR 4 stimulation. Most cytokines were detectable except IFN-α and IL-12p70, which were not produced above background by infants from any of our cohorts. The median cytokine concentration in response to TLR 4 stimulation for each individual cytokine response was consistently lowest for South African infants (Fig E1). Specifically, T_H_1 cytokine production in response to LPS revealed a low response pattern in South African infants, while the infants from the other 3 sites showed a higher median response. While the response overall appeared to have high variation between subjects even within a given site (note the larger spread of the CI), statistical differences were still detected (Table II). Dunn’s posttest identified that the greatest statistical difference for IFN-γ was found between Canadian and South African or Belgian children. Production of T_H_17-supporting cytokines following LPS stimulation also identified South African infants as having the lowest response to LPS. Similarly, production of proinflammatory cytokines and chemokines showed the weakest response in South African and the highest response in Canadian subjects; Dunn’s comparison identified the greatest statistical differences between South African versus Canadian and Ecuadorian infants. Production of IL-10 in response to LPS was again lowest in South African subjects.

#### TLR2/1 (PAM)

Overall, PAM stimulation resulted in the least obvious PCA cluster separation between sites for surface TLRs. However, only the response of the South African cohort strongly overlapped with the unstimulated samples, indicating an overall lower response, while the response of the subjects from other sites, primarily from Ecuador and Belgium, clearly clustered away from the unstimulated samples (Fig E2, *B*). With the exception of CXCL10, T_H_1-supporting cytokines were not produced above the background for subjects from any site in response to PAM (Fig E1). Dunn’s posttest of CXCL10 production following TLR2/1 stimulation revealed that infants from South Africa and Canada did not vary from each other in their response, but infants from both sites differed significantly in their response from that of infants from Belgium and Ecuador (Table II). The same relationship was found for the production of T_H_17-supporting cytokines, with infants from Belgium and Ecuador producing a higher median concentration than did infants from South Africa and Canada; however, only South African infants varied statistically from Belgian and Ecuadorian children. The production of proinflammatory cytokines was lower for infants from South Africa and Canada versus those from Belgium or Ecuador. The response of South African infants varied significantly from Ecuadorian and Belgian infants for all proinflammatory cytokines. The same statistical relationship was also detected for the production of IL-10.

### Cytosolic PRR responses: Similar responses in all cohorts

#### NOD2 and TLR2/1 (PGN)

Following PGN stimulation, the response of infants from all the sites, including South Africa, clustered tightly together (Fig 2, *C*). On closer inspection, we found that none of the T_H_1 cytokines were produced above background. Canadian and South African infants all produced significant levels of T_H_17-supporting innate cytokines following PGN stimulation, while Belgian and Ecuadorian infants responded weakly. Production of the proinflammatory cytokines and chemokines was also readily detected in infants from all sites, with differences between the 4 sites. Dunn’s comparison of IL-10 revealed strong significant variation between infants from South Africa (low) and those from Canada, Belgium, or Ecuador (Table II).

#### NOD2 (MDP)

Following stimulation with MDP, the response clusters visually overlapped with the unstimulated clusters for all sites, suggesting an overall very low response (Fig E2, *C*). T_H_1- and T_H_17-supporting, as well as anti-inflammatory, cytokines were not produced above background; the production of only CXCL10, TNF-α, CXCL8, and CCL4 were detectable. As with the other NOD ligand (PGN), the response of South African infants was more similar to that of infants from the other sites as compared with the TLR stimuli. Specifically, the production of CXCL10, TNF-α, CXCL8, and CCL4 in South African infants was similar to that in Canadian infants, while responses in both these groups were significantly lower than the responses of Belgian and Ecuadorian infants.

## Discussion

Our study represents the first test of the hypothesis that innate cytokine production in infancy following PRR stimulation varies across continents. By using a stringently controlled, robust, high-throughput innate immune phenotyping platform, we identified similarities as well as differences in innate immune response to PRR stimulation of samples collected from infants across 4 continents. When contrasting the infant innate cytokine response based on country, it emerged that the responses of South African infants for most stimuli were distinct from the responses of infants at the 3 other sites. This was notable both in the degree of separation of the clusters and in the consistency displayed across multiple stimuli. However, while the innate cytokine response to PRR stimulation in South African infants was found to be lower for nearly all parameters tested, it was similar to infants from the other sites for the NOD2 ligands PGN and MDP. This suggests that it was not an overall inability of South African infants to respond with cytokine production to PRR stimulation but that variation in the pattern of innate cytokine production following PRR stimulation in infancy varied by geographic region in response to the particular type of PRR stimulation. More specifically, the response to endosomal as well as cell surface PRRs varied by region, while the response to cytoplasmic stimuli was more similar among infants from different continents. This suggests differences in particular downstream signaling cascades in infants from South Africa versus the other sites.

Differences in innate immune status have been ascribed to variation in environmental exposures ranging from birth mode, feeding mode, infections, vaccination, and resource-rich versus resource-poor region of residence (reviewed in Kollmann et al^5^ and Kollmann^28^). However, based on these previous studies, the results of our current study—indicating striking differences between the innate immune response of South African infants versus those from Ecuador, Belgium, and Canada—were not predictable. Our work with Canadian infants revealed an overall steady increase from birth onward in the production of T_H_1-supporting innate cytokines following TLR stimulation, while TLR-induced anti-inflammatory and T_H_17-supporting innate cytokines progressively declined over the first 2 years of life.^5,12,19^ This was consistent with findings from other resource-rich regions of the world, for example, Belgium and the Netherlands.^29,30^ In contrast, with a similar experimental approach we revealed a decline of most TLR-induced innate cytokine responses in South African infants over the first year of life from a high at 2 weeks of age.^20^ Such decline over the first year of life was consistent with previous studies from The Gambia^20^ as well as Ecuador.^31^ Furthermore, cord blood mononuclear cells from Papua New Guinean newborns produce lower IL-6 and type-I IFN responses to TLR2 stimulation, and lower TNF-α responses to TLR4 stimulation as compared with Australian newborns.^32^ Over the first 2 years of their lives, Papua New Guinean infants then develop increasing IL-6 and IFN-γ responses to TLR2 and TLR3 agonists in parallel with sustained high IL-10 responses.^33^ Based on these data, it was hypothesized that infants born in resource-rich countries (ie, Australia) exhibit increased innate immune reactivity at birth than do infants born in resource-poor countries (ie, Papua New Guinea).^33,34^

The data presented here strongly argue that differences between regionally disparate groups in innate cytokine production following PRR stimulation were unlikely due to resource-rich versus resource-poor influence^33,34^ nor impacted by latitude,^35^ as infants from Ecuador (considered resource-poor) produced as much or more of any innate cytokine as did infants from Belgium or Canada (both considered resource-rich), and certainly more than did infants from South Africa (considered resource-poor). Furthermore, given that the subjects in our study from South Africa and Ecuador received Bacillus Calmette-Guérin around birth while subjects from Belgium and Canada did not, newborn Bacillus Calmette-Guérin immunization also appears unlikely to be the main driver for observed differences at age 2 years. Of note, Djuardi et al^36^ also found no clear effect of Bacillus Calmette-Guérin vaccination on the innate immune ontogeny. While the overall vaccination schedule was similar for all infants across the 4 sites, differences in vaccine composition (eg, acellular vs whole-cell pertussis) or exact age of receipt of vaccines differed; we thus cannot exclude that such variation in standard childhood vaccination might be responsible for our observed differences. We also interpret our data to indicate that differences in feeding mode (length of breast-feeding; breast- vs bottle-feeding etc) were unlikely major contributors to the differences we observed, as feeding mode differed vastly between and within sites. However, we can not exclude that feeding mode would not lead differences in innate immune development when comparing different feeding modes within a given population.^37^ Parasitic infections, which are common in South Africa and Ecuador early in life, but rare in Belgium and Canada, also were unlikely to offer a general explanation for the variation between populations we observed. However, we did not measure this directly in either the mothers or in our study subjects; thus, we cannot firmly exclude this possibility.^37^ Our data comparing secreted cytokine levels in the supernatant of whole blood cultures does not permit identification of the source or origin of observed differences in innate cytokine production; differences could thus be due to cell-intrinsic (eg, signaling mechanisms) or cell-extrinsic (eg, cell composition) factors, or a combination of both.

Differences in host genetic composition are known to influence innate immunity.^38^ We have recently shown that variation in innate immune responses can be influenced by single nucleotide polymorphisms within the PRR pathways and that the prevalence of these single nucleotide polymorphisms varies within different racial backgrounds.^39^ It is thus entirely possible that genetic differences (including variation in HLA) between our populations contributed to the differences in functional responses we measured between sites. The relatively small SD for the cytokine responses of each cohort suggests that genetic heterogeneity within each site was smaller than between sites. We also noted that the composition of enrolled subjects within each of our cohorts included a wide range of ancestral origin. We thus do not believe that differences in genetic background alone can explain the striking difference between responses to PRR stimulation of infants born and raised in South Africa versus our other 3 sites. We hypothesize that the particular constellation of microbiota in South African infants may contribute to our observed difference in innate immune phenotype. This hypothesis is based on the timing of the decline in innate responsiveness in South African infants (between 6 and 12 months of age) and the persistence of the lower innate responsiveness into adulthood.^20^ A stable human intestinal microbiota is established over the first year of life and then persists into adulthood.^40,41^

Infant mortality varies greatly between different regions of the world.^3^ In our 4 cohorts between 2005 and 2010, for every 1000 live births Belgium registered 5 deaths per annum before the age of 5 years, Canada 6, Ecuador 26, but South Africa 79.^42^ Low birth weight and gestational age often correlate with increased mortality^43^; the subjects in our cohort were of normal average birth weight, and the number of infants with lower gestational age at delivery in each cohort was similar between sites (Table I). These variables are therefore less likely to have caused the low innate immune response in only South African infants. Patterns of innate immune ontogeny in North American or European infants have been shown to correlate with particular age-dependent windows of vulnerability to specific infections.^5^ It is thus entirely possible that the overall lower response of South African infants at age 2 years to PRR stimulation reflects enhanced susceptibility to infection, and thus may be of clinical relevance.

The major limitation of our study is the small sample size; we thus cannot fully exclude the potential for a type 1 statistical error even after correcting for multiple comparisons. Our findings will need to be replicated in larger studies at the same and additional sites. Our recruitment strategy also was not representative of each population in its entirety. Notwithstanding these limitations, our data allow formulation of a molecular mechanistic hypothesis (“population-based differences exist in signaling downstream of surface and endosomal PRRs, but not of cytoplasmic PRRs”), and has identified possible relevant clinical ramifications (“differences in vaccine responses and infant mortality may relate to differences in innate PRR response”). Each of these hypotheses can now be tested in focused studies.

**Clinical implications**

Differences in innate immunity of 2-year-old infants across 4 continents correlate with variation in susceptibility to infectious morbidity and mortality.

## Figures and Tables

**Table I:** Demographics of the infants at each of the 4 sites

	Belgium	Canada	Ecuador	South Africa
N	14	20	43	20
Infant characteristics				
Mean age (mo), mean ± SD	24.7 ± 4.3	19.1 ± 0.8	26.7 ± 1.28	24.7 ± 0.6
Birth weight (g), mean ± SD	2996.2 ± 796.3	3339.6 ± 448.2	3475.1 ± 988.3	3018.4 ± 383.6
Birth mode (vaginal/c-section)	13/1	11/13	34/9	20/0
Gestational age, mean ± SD	38.4 ± 3.4	39.2 ± 1.5	38.9 ± 1.1	37.8 ± 2.4
Premature < 37 wk (% of total)	2 (14%)	1 (4.5%)	0 (0%)	3 (15%)
Weight (g), mean ± SD	13364.3 ± 1786.1	11190.9 ± 1392.5	11501.16 ± 1010.7	11205.0 ± 1300.7
Height (cm), mean ± SD	92.2 ± 4.6	82.2 ± 3.0	84.3 ± 2.5	84.4 ± 0.91
WAZ, mean ± SD	0.69 ± 1.2	−0.05 ± 0.9	−0.32 ± 0.93	−0.58 ± 0.95
LAZ, mean ± SD	1.56 ± 0.8	−0.30 ± 0.9	−0.78 ± 1.49	−1.07 ± 1.20
WLZ, mean ± SD	−0.18 ± 1.4	0.17 ± 1.0	0.16 ± 0.79	−0.03 ± 0.87

*LAZ*, Length-for-age *Z* score; *WAZ*, weight-for-age *Z* score; *WLZ*, weight-for-length *Z* score.

**Table II:** Statistical analysis of each cytokine per stimulation at all sites

	IFN-α2	IFN-γ	CXCL10	IL-12p70	IL-12p40	IL-6	IL-23	TNF-α	IL-1β	CXCL8	CCL3	CCL4	IL-10
Unstimulated													
Global	.003	.0956	<.0001	.6486	.2065	.0024	<.0001	.0002	.3932	.0153	<.0001	.3424	.838
SAF vs BLG			NS				∗	∗			NS		
SAF vs CND			†				‡	‡			‡		
SAF vs ECD			NS				NS	NS			NS		
BLG vs CND			∗				NS	NS			†		
BLG vs ECD			NS				NS	NS			NS		
CND vs ECD			‡				∗	∗			†		
R848													
Global	<.0001	<.0001	<.0001	<.0001	<.0001	<.0001	.0036	<.0001	<.0001	.0053	<.0001	<.0001	<.0001
SAF vs BLG	†	∗	†	NS	NS	‡		‡	∗		‡	†	‡
SAF vs CND	‡	‡	†	‡	‡	∗		‡	‡		†	‡	†
SAF vs ECD	‡	‡	‡	‡	‡	‡		‡	‡		‡	‡	‡
BLG vs CND	NS	NS	NS	∗	NS	NS		NS	NS		NS	NS	‡
BLG vs ECD	NS	NS	NS	‡	∗	NS		NS	∗		NS	NS	†
CND vs ECD	NS	NS	NS	NS	NS	†		NS	NS		†	NS	NS
pIC													
Global	<.0001	<.0001	<.0001	<.0001	<.0001	.1267	<.0001	<.0001	<.0001	.0041	<.0001	<.0001	.0031
SAF vs BLG	NS	NS	†	NS	‡		NS	NS	NS		NS	NS	
SAF vs CND	‡	‡	∗	†	NS		‡	‡	∗		‡	†	
SAF vs ECD	‡	‡	‡	‡	‡		NS	‡	†		‡	‡	
BLG vs CND	‡	†	NS	NS	‡		NS	NS	‡		†	NS	
BLG vs ECD	‡	†	NS	‡	NS		NS	†	‡		‡	‡	
CND vs ECD	NS	NS	NS	NS	‡		‡	NS	NS		NS	∗	
LPS													
Global	.0063	<.0001	<.0001	.1411	<.0001	<.0001	.0036	<.0001	<.0001	.0002	<.0001	<.0001	<.0001
SAF vs BLG		NS	†		‡	‡		∗	NS	†	‡	†	‡
SAF vs CND		‡	∗		‡	‡		‡	‡	NS	‡	†	‡
SAF vs ECD		†	‡		‡	‡		‡	†	†	‡	‡	‡
BLG vs CND		‡	NS		NS	NS		NS	‡	NS	NS	NS	‡
BLG vs ECD		NS	NS		NS	NS		NS	NS	NS	NS	NS	‡
CND vs ECD		∗	∗		†	NS		NS	‡	NS	NS	NS	NS
PAM													
Global	.0145	.6916	<.0001	.6243	<.0001	<.0001	.0001	<.0001	<.0001	<.0001	<.0001	<.0001	<.0001
SAF vs BLG			†		‡	‡	NS	‡	†	‡	‡	‡	‡
SAF vs CND			NS		NS	NS	NS	NS	NS	NS	NS	NS	NS
SAF vs ECD			‡		‡	‡	NS	‡	‡	‡	‡	‡	‡
BLG vs CND			∗		NS	∗	‡	NS	NS	‡	NS	NS	‡
BLG vs ECD			NS		NS	NS	NS	NS	NS	NS	NS	NS	NS
CND vs ECD			∗		NS	‡	NS	‡	†	‡	‡	‡	‡
PGN													
Global	.0174	.042	<.0001	.593	<.0001	<.0001	<.0001	<.0001	<.0001	<.0001	<.0001	.0044	<.0001
SAF vs BLG			NS		NS	‡	NS	NS	NS	‡	†		‡
SAF vs CND			NS		‡	NS	NS	‡	†	NS	NS		‡
SAF vs ECD			∗		NS	‡	‡	NS	NS	‡	‡		‡
BLG vs CND			∗		‡	‡	∗	NS	‡	‡	NS		NS
BLG vs ECD			NS		NS	NS	NS	NS	NS	NS	NS		‡
CND vs ECD			‡		‡	‡	‡	‡	‡	‡	NS		NS
MDP													
Global	.005	.2408	<.0001	.7072	.9201	.0254	<.0001	<.0001	.0006	<.0001	.0007	<.0001	.0477
SAF vs BLG			†				NS	∗		∗		NS	
SAF vs CND			NS				‡	∗		NS		NS	
SAF vs ECD			†				NS	‡		†		†	
BLG vs CND			†				NS	NS		†		NS	
BLG vs ECD			NS				NS	NS		NS		†	
CND vs ECD			‡				∗	†		‡		‡	

Note. The Kruskal-Wallis test for all 4 sites (global) was corrected for multiple comparisons by using the Bonferroni test (significant *P* value is *P* < .000595). *BLG*, Belgium; *CND*, Canada; *ECD*, Ecuador; *NS*, not significant; *SAF*, South Africa.

∗ Dunn’s post hoc test was applied to each site paring (statistical significance *P* value was <.05).

† Dunn’s post hoc test was applied to each site paring (statistical significance *P* value was <.01).

‡ Dunn’s post hoc test was applied to each site paring (statistical significance *P* value was <.001).
